# Silver, Its Salts and Application in Medicine and Pharmacy

**DOI:** 10.3390/ijms242115723

**Published:** 2023-10-29

**Authors:** Dominik Żyro, Joanna Sikora, Małgorzata Iwona Szynkowska-Jóźwik, Justyn Ochocki

**Affiliations:** 1 Department of Bioinorganic Chemistry, Medical University of Lodz, Muszynskiego 1, 90-151 Lodz, Poland; joanna.sikora@umed.lodz.pl; 2 Faculty of Chemistry, Institute of General and Ecological Chemistry, Lodz University of Technology, Zeromskiego 116, 90-543 Lodz, Poland; malgorzata.szynkowska@p.lodz.pl; 3 Medical University of Lodz, Muszynskiego 1, 90-151 Lodz, Poland

**Keywords:** silver (I), silver (I) salts, silver (I) complexes, antimicrobial activity, cytotoxicity, chemical stability

## Abstract

The healing properties of silver have been used since ancient times. The main aim of the study was to collect and review the literature on the clinical potential of silver, its salts and complex compounds. The second goal was to present an outline of the historical use of silver in medicine and pharmacy, taking into account the possibility of producing pharmaceutical drug forms on the premises of pharmacies. In the context of the growing resistance of microorganisms to available, widely used antibiotics, silver plays a key role. There is only one known case of bacterial resistance to silver—the *Pseudomonas stutzeri* strain, which naturally occurs in silver mines. The development of research in the field of coordination chemistry offers great opportunities in the design of new substances in which silver ions can be incorporated. These substances exhibit increased potency and often an extended antimicrobial spectrum. Silver-based compounds are, however, only limited to external applications, as opposed to their historic oral administration. Advanced studies of their physicochemical, microbiological, cytotoxic and genotoxic properties are ongoing and full of challenges. The improvement of the methods of synthesis gives the possibility of applying the newly synthesized compounds *ex tempore*, as was the case with the complex of metronidazole with silver (I) nitrate. Some of these experimental efforts performed in vitro are followed with clinical trials. The third and final goal of this study was to present the possibility of obtaining an ointment under the conditions of an actual pharmacy using silver (I) salts and a ligand, both of which are active substances with antimicrobial properties.

## 1. Introduction

Pure silver is scarce in the natural environment which is probably why it attracted attention of ancient people much later than gold. According to the Greek Chronicles, its discovery around 1300 BC is attributed to Ajakos. It was Hippocrates who had observed that this remarkable element has biological properties in the treatment and prevention of diseases. The Phoenicians kept water, wine, and vinegar in silver pots to prevent them from spoilage. The antimicrobial properties of silver were scientifically confirmed as early as in the 19th century, which laid the ground for the application of metal and its compounds in medicine. During World War I, silver compounds were used to prevent infections as antibiotics were not known then. As a standard solution, silver (I) nitrate was used which was later replaced with sulfadiazine ointment. With the discovery of antibiotics and sulfonamides, the interest in silver-containing drugs has temporarily decreased, but is now gaining new momentum. It was shown that silver (I) cation has a bactericidal, antiseptic, anti-inflammatory and astringent effect. It is a natural bactericidal metal that is effective against 650 species of bacteria with low reported resistance. This is advantageous over almost all antibiotics, the use of which steadily becomes more and more vain. The growing problem of microbial resistance to antibiotics and chemotherapeutics is a challenge for modern medicine. Every year, despite the advancement of treatment methods, growing health care standards and better public awareness of pharmacotherapy, the number of deaths caused by antibiotic-resistant bacterial strains increases [1]. This substantiates the search for new compounds with potential antimicrobial activity and turns attention of researchers to the potential use of precious metals in medicine. Research on elements such as copper, zinc, titanium, nickel, magnesium, gold and silver is thought to help develop promising methods of treatment against infections [1,2,3].

There are numerous studies reporting the cytotoxic and genotoxic potential of metal ions and their complexes towards normal and cancer cells. Greater stability of coordination compounds containing metal ions has also been demonstrated, compared to salts of these metals. These ions are incorporated in the structure of the compound and often exhibit biologically active effects [4,5,6,7,8,9]. Apart from the properties of the active substance itself, its clinical effect also depends on the method of administration, which is achieved by creating the appropriate form of the drug. Suitable level of fragmentation, well-selected solvent or substrate are fundamental factors to achieve desired outcome, i.e., proper absorption, concentration and, finally, the appropriate reaction in line with the mechanism of action of the medicinal substance.

## 2. Silver and Its Salts

Silver is a soft, malleable metal with a distinctive silver luster. It does not react with water or oxygen. Silver oxidation states [10,11,12] are presented in Table 1.

Most silver (I) salts, both inorganic and organic, are poorly soluble in water. Of these, only perchlorate, nitrate and fluoride have very good solubility; acetate, permanganate and sulfate have poor solubility (Table 2) [13].

Water, which is the most popular, most available and most economical reaction medium, enables electrolytic dissociation of salts, especially those that are well soluble in water. Silver is a heavy metal, and its highly soluble salts, such as nitrate or sulphate, undergo pronounced hydrolysis reaction in an aqueous environment according to the reactions:

AgNO_3_ → Ag^+^ + NO_3_^−^

Ag_2_SO_4_ → 2Ag^+^ + SO_4_^2−^

2Ag^+^ + 3H_2_O ↔ Ag_2_O + 2H_3_O^+^

Aqueous solutions of readily soluble silver (I) salts are therefore acidic. This is particularly important in the context of drug formulation. For instance, eye drops marked by too low pH may cause conjunctival irritation. Pharmacopoeic monographs indicate pH of the drops ranging from 3.5 to 8.5 [14,15].

## 3. Application of Silver and Silver (I) Salts in Medicine

Today’s scientists pay great attention to silver, although its preparations have been used for wound healing ever since ancient times [8]. Among metals, silver is particularly widely used in medicine and has a well-documented antimicrobial effect against Gram-positive and Gram-negative bacteria, fungi, protozoa and viruses [16,17]. The most common compounds of silver used as medicines are: silver (I) nitrate [18], silver sulfadiazine and silver sulfathiazole. Silver preparations containing colloidal silver are also frequently employed and include colargole, protargole and targezine (Table 3). Solid state silver (I) nitrate or in the form of concentrated (10–50%) aqueous solutions is used to cauterize tissues or to impregnate dentine. Silver (I) ions have also been shown to exert cytotoxic and genotoxic effects on various human cells by generating oxidative stress [19,20].

The usage of preparations containing silver has vastly changed over the years. Polish pharmacopoeias, starting with the first post-war edition as the second edition, which is a reprint of the pre-war edition from 1946, up until the most recent 12th edition, all contain monographs of silver preparations used in medicine and in pharmacy. The Polish Pharmacopoeia 2nd (FP II) edition contains the largest number of monographs of silver preparations, which are presented in Table 4 [21]. This article is a historical outline of the use of silver and its compounds over the timespan of nearly 80 years. In all countries of the former socialist bloc (Poland, the Czech Republic, Slovakia, East Germany, Bulgaria, Romania and others) national pharmacopeias were in force until these countries joined the European Union. It is impossible to provide only names consistent with the European Pharmacopoeia, because the monographs refer to specific preparations that were used historically. To the best of our knowledge, in addition to the Polish names, we have provided the most faithful translations in English. Since December 2006, i.e., since Poland ratified the Convention on the Development of a European Pharmacopoeia, the requirements of the European Pharmacopoeia (Ph. Eur.) are introduced directly into the Polish Pharmacopoeia. This process began with the 7th edition of the Polish Pharmacopoeia (volume I—2006, Supplement 2007), based on the materials of Ph. Eur. 5th edition.

The Polish Pharmacopoeia 3rd (FP III, published in 1954) no longer contains the monographs *Argentum colloidale*, *Argentum gelatinosum* or *Argentum nitricum fusum* [22]. Subsequent editions of the pharmacopoeia introduce a monograph on *Argentum colloidale*, a substance that is also used today. The Polish Pharmacopoeia 12th (FP XII, published in 2020) contains the Polish version of all materials published in the basic part of the European Pharmacopoeia 10.0 and in Supplements 10.1 and 10.2, as well as national sections, i.e., without equivalents in Ph. Eur. It presents the colloidal silver monograph as follows [18]:

ARGENTUM COLLOIDALE AD USUM EXTERNUM

Srebro koloidalne do użytku zewnętrznego

Silver, colloidal, for external use; Argent colloïdal pour usage externe

DEFINITION:
Colloidal, metallic silver containing protein.
Content: from 70.0% to 80.0% Ag (calculated on the dried substance).
PROPERTIES:
Appearance: green or bluish-black, metallic flakes or powder, hygroscopic.
Solubility: easily soluble or soluble in water, practically insoluble in ethanol (96%) and in methylene chloride.

In FP XII, the silver (I) nitrate monograph is still present despite the passage of nearly 70 years from the publication of the second edition of the Polish Pharmacopoeia. In the pharmacopoeias of other countries, we can find monographs of other silver preparations. In addition to silver nitrate and silver proteinate, Japanese Pharmacopoeia (JP XVII, published in 2016) contains an interesting monograph of silver proteinate solution composed of 0.22–0.26% silver alongside mint water and glycerin, the two of which are considered as *corrigens* [23]. The described pharmacopoeial medicinal product is used as an antiseptic mouthwash in the course of diseases associated with pharyngitis.

A silver preparation whose monograph has never been included in any of the FP editions is targetezine (*Argentum diacetyltanninoalbuminatum*, colloidal diacetyltanine-silver complex). However, the Therapeutic Guide to the Official List of Drugs (USL), an official document of the Polish People’s Republic from 1959, provides a description of the preparation [24]:

A substance with a bactericidal and astringent effect, both for oral and external application. It is used in catarrh of the conjunctiva and mucous membranes; in gonorrhea. Internally in gastric and duodenal ulcers. Externally in the form of solutions, ointments 0.5–4%. Internally, a 1–2% solution should be administered in tablespoons.

In addition to the above description, Therapeutic Guide to the USL enumerates other silver preparations available in pharmacies, described in FP III in the 1950s, which were also used orally according to the state of knowledge at the time. Table 5 presents the information published in the cited study (original wording was preserved).

The current, new editions of pharmacopoeias, along with the progress of pharmaceutical sciences, the development of toxicology, pharmacokinetics, pharmacodynamics and drug chemistry, provide the doses or concentrations of medicinal substances typically used and/or their maximum. This is important because over the years silver preparations have been moved to list A (very strong agents). FP XII contains the monographs of silver preparations and indicates the following values presented in Table 6.

As we can clearly see, the use of silver preparations internally has already been abandoned. This is related to the disease entity–argyria (*argyria*) [25] caused by unintentional absorption of silver compounds. The main symptom is a change in skin color to bluish-gray or slightly purple, especially in areas exposed to sunlight. Discoloration may cover some areas of the skin or its entire surface. This condition may be temporary and withdraw when absorption stops or the silver intake is discontinued. Therefore, the use of oral preparations is not currently recommended.

The first mention of the use of silver in medicine dates back to ancient times. It is probable that Hippocrates used silver preparations to treat ulcers and sores in order to accelerate wound healing. Soluble silver (I) compounds, such as silver (I) nitrate, were first used empirically as blood purifiers in 702–705 AD [26]. Later, silver (I) salts were used as antibacterial agents to treat infectious diseases, including syphilis and gonorrhea, brain infections, epilepsy, mental illness, nicotine addiction and gastroenteritis [17]. The widest use of silver in medicine was reported in the 1880s. Then, the first silver plate was implanted during cranial surgery, followed by silver eye drops being used. The nitrate solution was introduced into medicine to prevent childhood blindness and reduce the number of cases of *ophthalmia neonatorum* [27]. Obligatory ophthalmic prophylaxis in newborns with silver (I) nitrate drops, as in the Credé method, was adopted in many countries around the world until the 1970s, and in some areas it is still a routine part of the perinatal period [28,29]. Over the years, another application of silver (I) preparations appeared. Their use has been extended to treat corneal ulcers, interstitial keratitis, blepharitis and cystitis [30]. Other silver preparations that were used in medicine in the last century were registered under various trade names. Some of these include: Albargin^®^ (*Argentum gelatinosum*), Choleval^®^ (by Merk and Co. in New York, NY, USA), Ammargen^®^, Argoflavin^®^ (a combination of tripaflavin with silver nitrate, which exhibited a synergistic effect of the two components—used as bactericide for topical applications and for intravenous injection), Poviargol^®^ (*Protargole*) [31,32,33,34,35].

Recently, the anticancer effect of silver (I) nitrate associated with the induction of apoptosis in H-ras 5RP7 cells has been discussed [36]. The research results also prove that the anticancer effect of silver (I) compounds does not apply only to its well-dissociated salts, but also to silver (I) complex compounds [37]. Silver, which is a transition metal, has the ability to form coordination compounds. This has been the subject of research for many years, because many complex compounds where silver is coordinated can become potential therapeutic agents due to the unique biological effect of the silver (I) ion. It is also extremely desirable that the ligands, as structural parts of the silver (I) coordination linkage, show proven clinical effectiveness, such as, for example, metronidazole (MTZ) or 4-hydroxymethylpyridine derivatives. As a result of the action of the silver (I) ion and the ligand, at least a synergistic effect should be expected, however, studies indicate a hyperaddition synergism [2,38,39]. When silver comes into contact with microorganisms, there is an immediate disruption of the cell wall, which later leads to the death of these organisms. It has been proven that silver affects the metabolic behavior of bacteria, viruses and eukaryotic microorganisms. It has been suggested that silver (I) ions modify their pathogenic activity by interacting with microbial electron transport systems, cell membranes and the DNA binding mechanism. Silver has a broad spectrum of activity and is less likely to cause microbial resistance than conventional antibiotics. In addition, the antibacterial effect of silver can be enhanced by its combination with other antimicrobial agents, which should be taken into account [40,41,42]. The method of synthesizing the silver complex with 4-hydroxymethylpyridine is a patented invention [43,44].

In medicine, silver is used not only in the form of dissociating salts, but also as nanoparticles (colloidal silver) [45]. Silver owes its antibacterial and antifungal properties solely to its ionic Ag+ form, which, however, is quite unstable and can be easily inactivated by improper complexation and precipitation, or it can be transformed into the metallic Ag (0) form lacking healing properties [46]. Pure metal continuously releases small amounts of ions that have an antibacterial effect on the metal surface [45]. The standard potential of the Ag+/Ag system is +0.7992 V. Oxidation to the Ag+ ion is a slow process under normal conditions and leads to low effective concentrations of silver. Therefore, metallic silver is used in the alloys to coat implants or sutures [47,48]. Silver (I) salts differ in terms of their solubility and are thus capable of generating silver ions to varying degrees. The high solubility of silver (I) salts leads to a high local concentration of silver, which translates to high antibacterial activity, but also high toxicity. The solubility and toxicity of silver (I) salts depend on many external factors, e.g., they change depending on pH. Therefore, each medicinal product containing silver (I) salts requires thorough clinical studies to assess the actual concentration of silver (I) ions [28].

The synthesis of silver in the nanoparticle (colloidal) form consists in the reduction of the soluble silver (I) salt by a reducing agent such as citrate, glucose, ethylene glycol or sodium borohydride [49]. The decisive role is played by the addition of stabilizing compounds that prevent the growth and aggregation of the formed silver nanoparticles [38]. Reproducible synthesis of silver nanoparticles in laboratory conditions is difficult and depends, among others, on the concentrations, reducing agent, temperature and the presence of additives. Moreover, the morphology of the obtained particles is not always stable. Often, synthesized silver nanoparticles tend to aggregate after a few hours or days if colloidal stability is insufficient [45,49].

Silver nanoparticles (Ag-NPs) are capable of creating nanostructures and are therefore used not only in medicine, but also in biotechnology, electronics, environmental remediation, biosensors, agriculture and the food industry. For technical applications on an industrial scale, Ag-NPs are produced mainly using physicochemical techniques: gamma radiation, electrochemical methods, chemical reduction and others. Alternatively, a so-called green synthesis can be used, which reduces production cost and prevents introduction of toxic residues into the natural environment. Studies suggest that biogenic Ag-NPs are even less toxic in vivo than chemically synthesized nanoparticles [50]. Ag-NPs can be synthesized biologically using microbes such as *Bacillus subtilis* and *B. licheniformis* (Gram-positive bacteria), *Escherichia coli* (Gram-negative bacteria), fungi, yeasts and viruses [46,51]. In addition, due to the richness of alkaloids, saponins, tannins, vitamins, phenols and terpenoids in organic matrix, the synthesis of Ag-NPs takes advantage of plants, plant products and algae as reducing biological agents, providing an inexpensive, one-step procedure [52]. Novel silver nanoparticles are attractive as antimicrobial agents due to their ability to function on the surface and the ability to cleave disulfide bonds. Ag-NPs act on bacteria, fungi and viruses in a shape-dependent manner. As particle size decreases, the percentage of surface atoms increases, forming many unsaturated bonds due to the absence of adjacent atoms. As a consequence, Ag-NPs possess unstable atoms with high surface energy. This type of structure provides multiple contact adsorption sites and reaction points that can be further modified [53].

Scientists also report the toxicity of Ag-NPs to living organisms as an important issue. The work of El-Samad and co-authors clearly indicates overproduction of reactive oxygen species (ROS) inside cells in beetles. In this study, DNA impairment and apoptosis of midgut cells were assessed using alkaline comet test and flow cytometry, respectively. It has been confirmed that silver nanoparticles cause physiological, genotoxic and ultrastructural anomalies in tissues [54]. Ag-NPs can also cause impairment of mitochondrial function as early as before their penetration and accumulation in the mitochondrial membrane, as presented in the work of Akter et al. [55].

Metallic silver is usually inert, but after implantation in the presence of tissues, it is ionized under the influence of oxygen, moisture and body fluids, releasing biologically active silver ions (Ag+), which bind to thiol groups (-SH), anionic protein ligands and cell membranes of bacterial cells [45]. What underlies the basis of the antimicrobial activity of silver is the ability of Ag+ to penetrate bacterial cell walls through pinocytosis, causing an increase in cellular oxidative stress in microorganisms—denaturing and inactivating proteins, as well as metabolic enzymes, which leads to growth inhibition [56,57]. Ionic silver (I) also has the ability to bind to the microbial genome (DNA or RNA), which inhibits replication of nucleic acids and prevents multiplication of microorganisms [58].

The latest discovery of Ag-NPs as biocides is related to their effectiveness as antivirals targeting infectious diseases such as: SARS-CoV, Influenza A/H5N1, Influenza A/H1N1, Herpes simplex virus types 1 and 2, Human parainfluenza virus type 3, dengue virus, HIV-1, hepatitis B virus and new encephalitis viruses. The exact mechanism of action of Ag-NPs as antivirals has not yet been fully elucidated [55]. In general, silver nanoparticles are able to reduce virus infectivity, probably by blocking virus-cell interaction, which may depend on the size and zeta potential of silver nanoparticles [59]. In vitro studies have shown the effectiveness of silver nanoparticles modified with oseltamivir in reducing influenza glycoproteins and preventing DNA fragmentation, chromatin condensation and caspase-3 function, which enabled to effectively mitigate H1N1 infection [60,61]. Recent studies have revealed suppression of human parainfluenza 3 (HPIV-3) replication through the use of Ag-NPs [62].

The anti-inflammatory effect of silver (I) nitrate or nanocrystalline silver has been experimentally confirmed in the treatment of wounds, treatment of allergic contact dermatitis and ulcerative colitis [63,64,65]. Experimental studies have shown a reduction in inflammation after the use of nanocrystalline silver, which was associated with lymphocyte apoptosis, decreased expression of pro-inflammatory cytokines and reduced gelatinase activity [26].

Research on the use of new silver preparations in combination with other active substances is also developing quite dynamically in ophthalmology. The widest and best-known example of silver used in medicine is sulfadiazine (AgSD), which became a topical antibacterial agent for the treatment of burns and fungal keratitis [66,67,68]. The profile of AgSD also shows a strong antibacterial potential against *E. coli, S. aureus, Klebsiella* spp. and *Pseudomonas* spp. [58].

## 4. Silver (I) Complexes

Introduction of cis-platinum into medicine marks the beginning of scientists’ interest in complex, specifically designed and biologically active compounds of noble metals. The historic moment happened more than 40 years ago. Cis-platinum was approved for use in chemotherapy for testicular and ovarian cancer by the US Food and Drug Administration (FDA) on 19 December 1978 [69,70,71] and in the UK (and several other European countries) in 1979 [72].

The toxicity of silver can be assessed as low. This metal does not play any biological role, but on the other hand, it causes argyria—probably as a result of transformation into unstable silver (I) chloride [73,74,75,76]. Low toxicity to the human body is undoubtedly an advantage, which is why the search for new silver (I) complex compounds is gaining momentum, what is similar to the search for an alternative to cis-platinum and its analogues (carboplatin, oxaliplatin), which would cause less side effects and would not show resistance.

It has been shown in numerous works that silver (I) complexes, apart from their effect on microorganisms, also have anti-cancer properties [77,78,79,80,81,82]. Selective cytotoxicity against various cell types depends on the type of ligand bound to silver (I) ions, which in turn is closely related to the stability of the complexes and the hydrophilic-lipophilic properties of the complexes formed by the ligand [78,83,84]. It was, in fact, the aim of many studies to determine whether the toxicity of AgNO_3_ in complexes is reduced by the synergistic effect caused by the presence of ligand molecules, for example MTZ or 4-hydroxymethylpyridine [40,44].

Many teams worldwide have conducted numerous studies that have shown not only the cytotoxic and genotoxic potential of metal ions and their complexes against normal and cancer cells, but also greater stability of metal ion complexes compared to free salts of these metals [4,5,6,7,8,9,85,86,87].

Heterocyclic compounds hold promise in these efforts because of the presence of nitrogen, sulfur or oxygen atoms in their structure, which are capable of coordinating metal ions. N-heterocyclic ligands, especially polypyridines, have been extensively studied in combination with many metals in the search for new anti-cancer drug candidates [88]. The bonds of N-Ag-N are the most important in this context.

**Figure 1:**
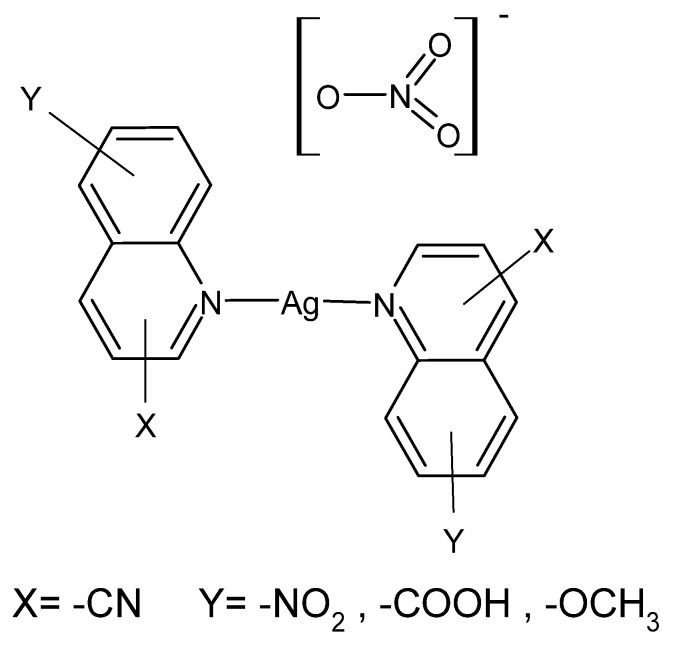
Coordination compounds of quinoline derivatives with silver (I).

Quinoline derivatives described in the literature have been tested for activity against 15 different multidrug-resistant bacterial strains isolated from diabetic foot ulcers and their antimicrobial activity was compared with the reference drug—AgSD. [Ag(8-nitroquinoline)_2_]NO_3_·H_2_O was the most active compound in this series, and its effectiveness turned out to be greater than AgSD against all tested bacterial strains. In turn, [Ag(5-nitroquinoline)_2_]NO_3_ gave better bactericidal results than AgNO_3_ against standard non-resistant bacterial strains *S. aureus*, *P.aeruginosa*, *Proteus mirabilis* and *Streptococcus pyogenes* [89]. Compounds are presented in Figure 1.

**Figure 2:**
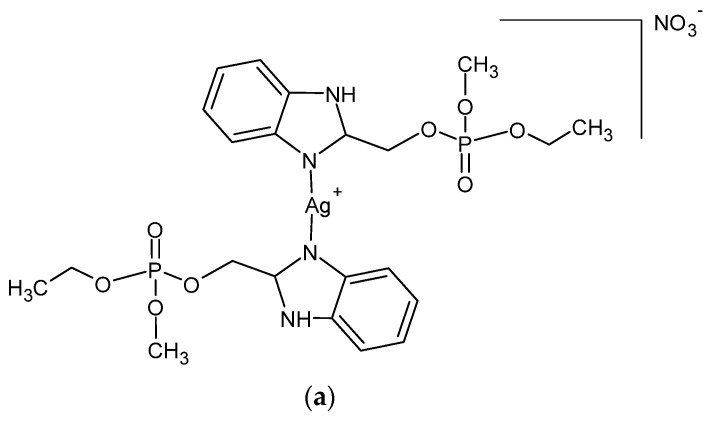

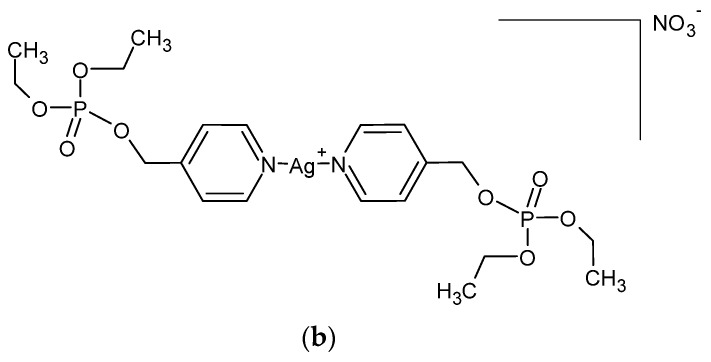
Coordination silver (I) compounds with benzimidazole (**a**) and pyridine (**b**) phosphate derivatives.

A series of Ag (I) complexes with phosphate derivatives of benzimidazole and pyridine (Figure 2) also shows antifungal activity against *C. albicans*. Among them, [Ag(2-bimOpe)_2_]NO_3_ (2-bimOpe = 1H-benzimidazol-2-ylmethyldiethylphosphate) was highly effective against *P. aeruginosa* and methicillin-resistant *S. epidermidis*, while the free ligands showed no activity [41].

Studies of antiproliferative activity using various types of cancer cells were also conducted with a complex composed of one of the valine esters as its ligand. Complex compound with formula: [Ag(L)(PR_3_)]^+^ (where L = valine N-(4-pyridinylcarbonyl)-methyl ester, PR_3_ = PPh3, PPh_2_Py) in which the pyridine nitrogen atom coordinated silver (I), induced the death of the examined cells by apoptosis [90].

Designing new drug candidates involves, among other things, attempts to modify already discovered molecules, followed by research to improve parameters such as: their ability to target specific bacterial strains, increasing or decreasing their cytotoxic effect, acquiring a new effect, mitigating their side effects, etc. A perfect example is the group of barbiturates, which includes many members, but each compound has different properties due to the introduction of specific substituents that affect both their character as a drug acting on the human body, as well as the direction or strength of action. In research work, molecules that have an established position as medicinal substances are used, but also new ones are being designed. The oldest of the silver (I) coordination compounds—silver salt of sulfadiazine (Ag-SD) and silver (I) coordination compound with MTZ should be mentioned here.

**Figure 3:**
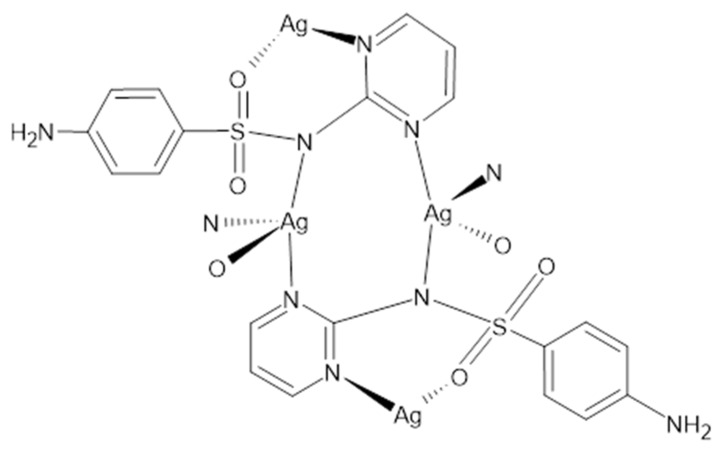
Structure of the silver (I) sulfadiazine salt.

**Figure 4:**
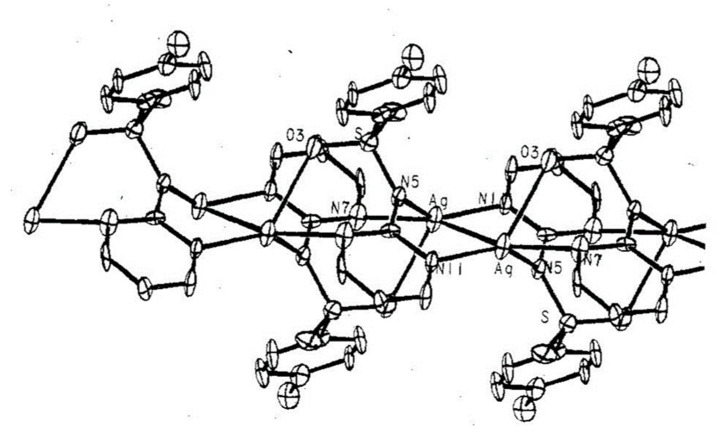
X-ray structure of the silver (I) sulfadiazine salt.

Sulfadiazine (4-amino-N-pyridin-2-yl-benzenesulfonamide) belongs to the group of antibacterial drugs—sulfonamides. It is currently on the World Health Organization’s List of Essential Medicines [91]. Used together with pyrimethamine, it is the drug of choice for toxoplasmosis [92]. It is also a second-line oral treatment against otitis media, prevents rheumatic fever, chlamydia and infections by *Haemophilus influenzae*. Sulfadiazine was approved for medical use in the United States in 1941. AgSD, which is the silver salt of sulfadiazine, from the chemical point of view, is a coordination compound, the structure of which is shown in Figure 3. The compound was discovered in the 1960s and is now on the World Health Organization’s List of Essential Medicines, although it is rarely used anymore. X-ray structural studies performed more than 40 years ago [93] led to the development of the compound shown in Figure 4. The drug has an antibacterial effect and is intended for the topical treatment of superficial wounds, such as burns. It is usually applied in the form of ointment or 1% water suspension. It is used on wounds in the healing phase or after skin grafts. It prevents the development of a wide spectrum of bacteria and yeasts, and when applied on the skin, it exerts an effect from both sulfonamide and a silver cation released after administration. AgSD is not soluble in water. Silver binds to exudate proteins in the wound area, releasing sulfadiazine, up to 10% of which is absorbed. Long term use may lead to localized argyria considering the silver content of this drug.

**Figure 5:**
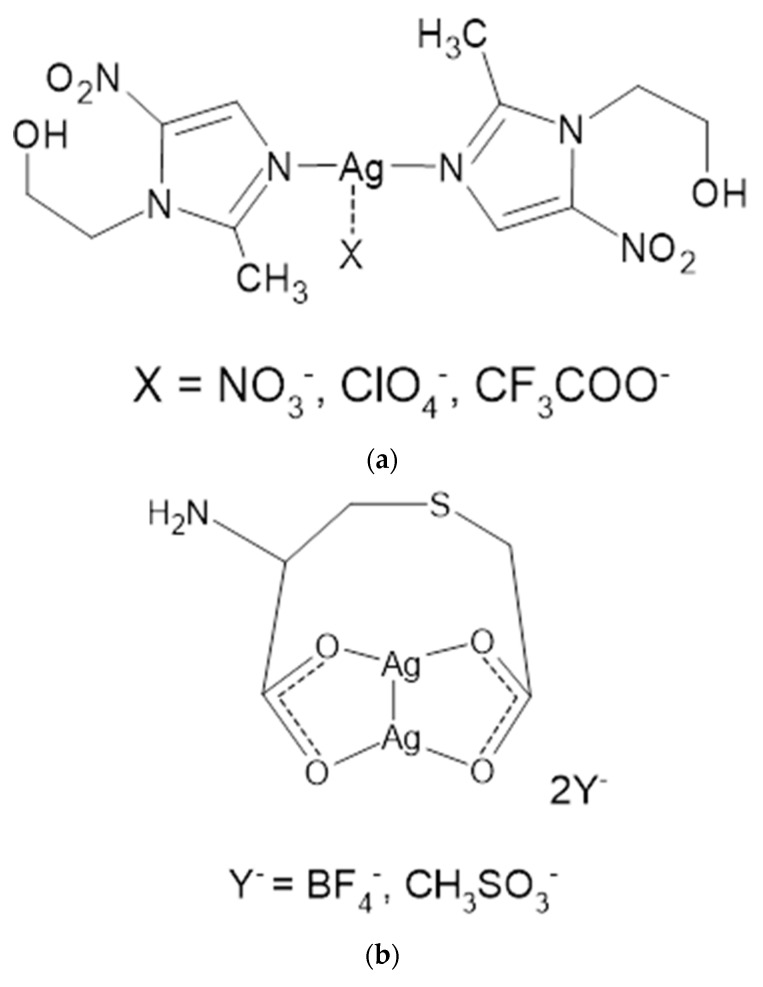
Structures of MTZ complex compounds of silver (I) with counterions: nitrate, perchlorate and trifluoroacetate (**a**); with tetrafluoroborate and methanesulfonate (**b**).

MTZ, due to the presence of a pyrazole nitrogen atom, is able to form coordination compounds with metals, including the silver cation. Taking advantage of these properties and the fact that MTZ is a well-established and proven drug, water-soluble complexes of metronidazole with silver were synthesized using several silver salts as substrates [40]. New MTZ coordination compounds with silver (I) were synthesized in the form of monomers of the general formula [Ag(MTZ)_2_]^+^X^−^, where X = NO_3_^−^, ClO_4_^−^, CF_3_COO^−^ (Figure 5a) or dimers, [Ag_2_(MTZ)_4_]^2+^2Y^−^, where Y = BF_4_^−^ and CH_3_SO_3_^−^ (Figure 5b). The microbiological properties of the salt were determined on Gram-positive (*S. aureus, S. epidermidis*) and Gram-negative (*P. aeruginosa, E. coli, Proteus hauseri*) bacterial strains and on *C. albicans* yeast. Each of the compounds showed significant antibacterial activity against Gram-positive bacteria, higher than AgSD, as a reference drug. The importance of the counterion was also assessed in antibacterial tests. The most potent Ag-MTZ complex was containing methanesulfonate counterion, which also inhibited the growth of yeast *C. albicans* at a concentration 3-fold lower than what was reported for AgSD. In addition, complexes containing tetrafluoroborate and perchlorate as counterions have also been characterized as effective antibacterial agents against the Gram-negative bacteria tested.

**Figure 6:**
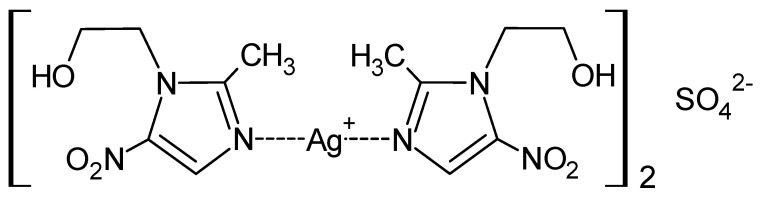
Structure of MTZ complex with silver (I) sulphate.

Modification of the method of synthesis of the MTZ complex with silver nitrate (I) to adopt *ex tempore* approach opened a way to the development of the formulation of the drug form [20,94]. Further, while the effect of the counterion in complexes made of metronidazole with various silver salts had previously been investigated, this work was far from completion. Ultimately, the synthesis of the MTZ coordination compound with silver (I) sulphate was accomplished, as presented in Figure 6 [86].

The development of a one-step synthesis of metronidazole complex of with silver (I) nitrate allowed our team to create the formulation of ointment for external use, ophthalmic ointment, eye drops and water-based gel for external use [94]. Drug forms have been produced both in laboratory conditions and in the operating conditions of an actual public pharmacy. Active and auxiliary substances, which served as pharmaceutical raw materials for the recipe, were purchased from pharmaceutical wholesale distributors.

An ointment was prepared according to prescription:

*Rp.*
*Argenti nitrici 1.0*
*Metronidazoli 2.0*
*Aquae destillatae q.s.*
*Eucerini S ad 100.0*
*M.f.Ung. D.S.* externally 2 times a day

and in accordance with generally accepted standards used in pharmacy practice [14,18]. A solution of the substance prepared *ex tempore*, containing a complex of metronidazole with silver (I) nitrate, was added in small portions to the appropriate amount of the base (Eucerin S). As a result of the procedure, an emulsion ointment was obtained.

**Figure 7:**
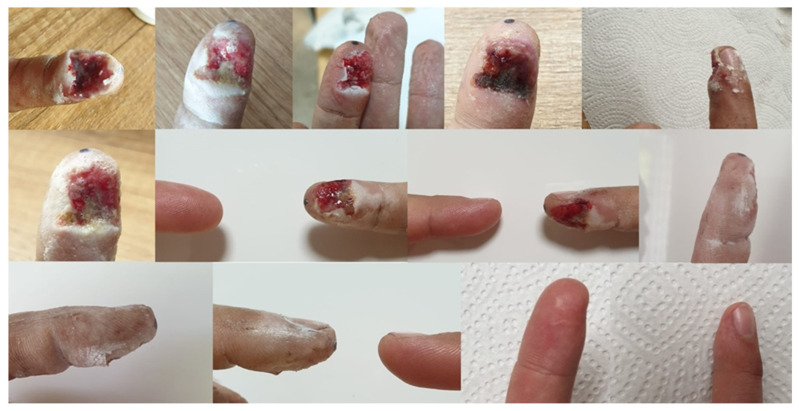
The process of healing the wound after cutting off the tip of the index finger, documented with photographs. The last two photos show the current condition—one year after the end of treatment [95].

**Figure 8:**
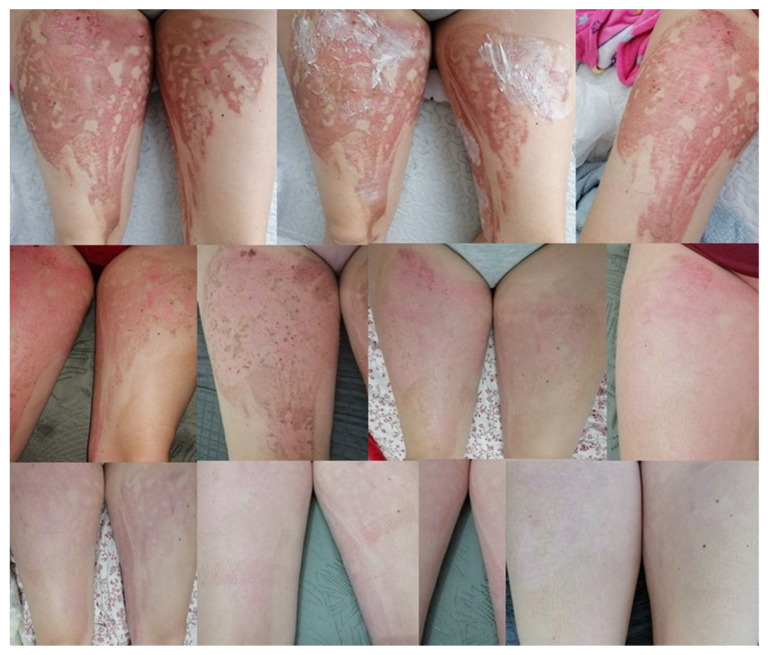
The process of treating post-burn lesions documented with photographs. The last photo is the current condition—a month after the end of treatment [95].

The ointment prescribed by doctors, prepared according to the formulation, was used on several patients. The treatment gave good results, with visible healing when applied twice a day for a period of a week up to a month [95]. Below, two cases were shown. Patient 1—shearing of the tip of the index finger by a grinding disc. The changes that occurred during the therapy are presented in Figure 7. Patient 2—burns of the lower limbs (both thighs) on the outside with water at a temperature of approximately 90 °C. The progress of the therapy is presented in Figure 8.

It should be noted that a drug containing a complex of metronidazole with silver (I) nitrate, prepared according to the doctor’s recommendation, gives better wound healing results. According to previous studies, this complex has lower MIC and MBC values for anaerobic bacteria than sulfadiazine silver (I) salt—one of just two silver complex structures registered as drugs at the present time. The latter was also used as a reference drug in the earlier study [86]. Synthesized complex [Ag(MTZ)_2_]_2_SO_4_ · 5H_2_O, in contrast to [Ag(MTZ)_2_ NO_3_], shows a polymeric structure. It is worth noting that Ag_2_SO_4_ was used for the first time, which, due to its chemical and physical properties, had not been used to date for the synthesis of complexes. An obtained compound exhibited desired chemical and biological properties. The probable synergism of both components of the complex—metronidazole causing DNA damage and silver binding to thiol groups (-SH)—is responsible for the anticancer and bactericidal effect. [Ag(MTZ)_2_]_2_SO_4_ and [Ag(MTZ)_2_NO_3_] can be considered as compounds showing a unique risk-benefit relationship resulting from low cytotoxicity against fibroblasts (Balb/c 3T3) and from their antimicrobial activity.

Many research teams continue to work on further benefits of the medical potential of silver and its compounds. Candido et al. conducted research assessing the antiproliferative effect of the silver (I) complex with nimesulide (AgNMS) in the SCC4, SCC15 and FaDu SCC lines. The use of this complex was effective and safe in the treatment of skin squamous cell carcinoma (SSCC) in mice and may be viewed as a potential and safe agent for the topical treatment in humans [96]. TPGS/Mup-Ag is a d-α-tocopherol polyethylene glycol 1000 succinate (TPGS) modified mupirocin and silver complex. Silver ions are released from the mupirocin-Ag complex (Mup-Ag) and thus may exert a synergistic antibacterial effect with mupirocin. Studies of Sun and co-authors with mupirocin-resistant *Staphylococcus aureus* (MuRSA) may contribute to the development of therapeutic agents for antibiotic-resistant bacteria and offer new ideas for silver-based bactericides [97]. Rao et al.’s team developed a cotton fabric impregnated with SNPNPs (SNPCFs), which remain photoneutral and exhibit long-term antimicrobial activity through surface modification with a silver nitroprusside complex. SNPCFs have been shown to have excellent wound healing properties when applied topically to the skin, so they have great potential as new antimicrobial and wound healing agents in the future [98]. In 2021, 2-(3, 5-dimethyl-1H-pyrazol-1-yl)-acetophenone oximes and their complexes with silver were synthesized and their pharmacological exploration was carried out. Six of them displayed excellent antibacterial activity against four bacterial strains with using Ciprofloxacin as a standard drug [99]. Research is also being carried out on HT-29 colorectal adenocarcinoma cells. The cells were treated with two new complexes: silver (I) diphenyl-2-pyridylphosphine]Br and [silver (I) 4-(dimethylamino)phenyldiphenylphosphine]Br. The first one has a greater inhibitory effect, significantly targets mitochondria and is selective towards HT-29 cells. It may be considered as a metal-based drug candidate that could be effective against colorectal cancer [100]. The research on silver-amino acid complexes is also important. Antibacterial, anticancer and selectivity properties were assessed with new complexes AgVal and AgAsp. They have been very carefully characterized. Both compounds inhibit the growth of cancer cells and pathogens by cleaving the genome [101].

## 5. Conclusions

The disastrous resistance of bacteria to the modern antimicrobial agents is largely caused by incorrect prescriptions, overuse or misuse of antibiotics. We therefore turn back to already known solutions that could bypass this problem. Rather than looking for new chemical compounds with desired biological properties, researchers often modify the structures of long-established molecules or create new adducts from substances with well-documented effects. Many previous studies have determined the chemical and biological properties of coordination compounds made up of imidazole derivatives and transition metals, including silver. The results of these works are valid for today’s drug development and their application could eventually progress available treatments. Systemic use of preparations containing silver in the form of salts or coordination compounds may cause generalized or local argyria. However, this does not exclude external applications, i.e., administration onto body cavities. Such an approach allows to confine high local concentration of the biologically active substance. The work on the formulation of drugs containing silver complexes is still ongoing. These compounds are tested for how they promote wound healing, reversal of skin lesions or their beneficial effects on the organ of vision.

Further research on compounds containing silver in ionized form (Ag^+^), formed as a result of the dissociation of both salts and silver complexes, should be carefully monitored. Tests should consider their cytotoxicity towards normal cell lines (e.g., human fibroblasts) and cancer cells as well as their specific properties, i.e., antibacterial, antiviral, antifungal, etc. Many of the azole derivatives exhibit these characteristics (e.g., metronidazole, tinidazole, ketoconazole, voriconazole, fluconazole, miconazole). Combining azoles into complexes with silver salts allows not only to reduce the MIC and MBC or IC_50_ values, but also often expands the spectrum of action of a drug. Biocompatibility assessment is also necessary (PT and APTT tests, erythrotoxicity assessment). Due to application limitations, research should be conducted primarily on skin melanoma cells and other types of cancer cells whenever external application of products containing silver complexes is possible. Silver complex compounds with azole structures offer great promise in dermatology (ointments, solutions for external use, gels, creams), ophthalmology (eye drops, eye ointments and gels), dentistry (dental fluids and gels), otorhinolaryngology (drops or ointments for nose, ears, gargles) and gynecology (creams, ointments and vaginal pessaries).

## Figures and Tables

**Table 1:** Silver oxidation states (the most important ones were bolded).

Oxidation State	Electronic Configuration	Examples of Compounds
Ag ^0^	d^10^s^1^	rare; Ag(CO)_3_ in 10 K
**Ag ^I^**	**d^10^**	**Ag_2_O, [Ag(OH)_2_]^−^ (aq.), [Ag(H_2_O)_4_]^+^, AgF, AgCl, ** **Ag^+^ salts e.g., AgNO_3_, Ag_2_SO_4_, Ag_2_S. Ag(CN)_2_^−^** **and other complexes**
Ag ^II^	d^9^	AgF_2_, [Ag(C_5_H_5_N)_2_]^+^, AgSO_4_, Ag ^I^ and Ag ^III^ are in AgO (not Ag ^II^)
Ag ^III^	d^8^	rare; [AgF_4_]^−^, [AgF_6_]^3−^

**Table 2:** Solubility of silver (I) salts in water.

Silver (I) Salt	Chemical Formula	Solubility in Temp. 25 °C (g/100 g H_2_O)
Perchlorate	AgClO_4_	500.0
Nitrate	AgNO_3_	257.0
Fluoride	AgF	100.0
Acetate	CH_3_COOAg	1.11
Permanganate	AgMnO_4_	0.9
Sulfate	Ag_2_SO_4_	0.83
Nitrite	AgNO_2_	0.42
Bromate	AgBrO_3_	0.16
Salicylate	C_6_H_4_(OH)COOAg	0.095 (in 23 °C)
Iodate	AgIO_3_	0.044
Dichromate	Ag_2_Cr_2_O_7_	0.0083 (in 15 °C)
Chromate	Ag_2_CrO_4_	0.0035
Carbonate	Ag_2_CO_3_	0.0033
Citrinate	C_6_H_8_O_7_Ag_3_	0.00284
Phosphate	Ag_3_PO_4_	0.000644
Chloride	AgCl	0.000193
Stearate	CH_3_(CH_2_)_16_COOAg	0.000065
Sulphide	Ag_2_S	0.000014
Bromide	AgBr	0.0000135
Iodide	AgI	0.00000026
Cyanide	AgCN	0.00000023

**Table 3:** Silver preparations used in medicine.

Composition	Name—Dosage Form	Concentration	Application
Silver nitrate (I) with potassium nitrate	Lapis—pin	97%	Tissue cauterization
Silver nitrate (I)	Mova Nitrat Pipette ^®^—eye drops	10 mg/mL	Credé’s prophylaxis
Silver nitrate (I)	Component of pharmaceutical products	-	Pharmaceutical
Silver (I) sulfadiazine	Dermazin ^®^, Silvadene ^®^—creme	10 mg/g	Burns of all degrees and sizes
Silver (I) sulfathiazole	Argosulfan ^®^—creme	20 mg/g	Burns of all degrees and sizes
Finely divided silver (0) with protein or gelatin	Colargole, *Argentum colloidale,* *Collargolum, Corgolum*— component of pharmaceutical products	-	Pharmaceutical
Organic silver (I) complex with protein	Protargole, *Argentum proteinicum, * *Protargolum, Prorgolum*— component of pharmaceutical products	-	Pharmaceutical
Diacetyl Tannin Silver (I) Proteinate	Targezine, *Targesinum*, *Argentum diacetylotannicum albuminatum*— component of pharmaceutical products	-	Pharmaceutical

**Table 4:** Monographs of silver preparations FP II.

Name According to the Monograph in FP II	Polish Name	Synonyms FP II	Description (Original Wording)	Form and Properties (Original Wording)
***Argentum colloidale***	Srebro koloidalne	Corgol, Collargol	*Argentum colloidale* should contain not less than 70% Ag (atomic weight 107.9), the rest is: protein or gelatin and a small amount of silver chloride	Dark gray flakes with a metallic sheen, odorless, with a faint metallic taste. Colloidal silver slowly dissolves in the water. *Argentum colloidale* solution in 50 parts of water is opaque, and in reflected light—cloudy; becomes transparent when diluted with plenty of water, in reflected light, however, it is still cloudy.
***Argentum gelatinosum***	Żelatynian srebrowy	Argentum gelatosatum, Gelargin, Albargin, Gelatynian srebrowy	Argentum gelatinosum should contain no less than 14.6% and no more than 15% Ag (atomic weight 107.9)	Yellowish, fine, shimmering, odorless powder with a salty-insipid taste. Argentum gelatinosum is easily solublein water giving neutral or slightly acidic solutions.
***Argentum nitricum***	Azotan srebrowy	Argenti nitras, Argentum nitricum crystallisatum, Lapis infernalis, Lapis, Kamień piekielny	The preparation should contain no less than 99.6% AgNO_3_	Colourless, odourless, transparent belts of the rhombic system. Silver nitrate dissolves in 0.5 part of water, in 15 parts of alcohol; very sparingly soluble in ether and glycerol. M.p. around 208 °C. Silver nitrate at 208 °C has the form of a yellowish liquid, which after solidification forms a white, crystalline mass; heated at a temperature higher than 208 °C, it gradually decomposes, releasing brown fumes.
***Argentum nitricum cum*** ***Kalio nitrico***	Azotan srebrowy z azotanem potasowym	Argenti nitras et Kalii nitras, Argentum nitricum fusum mitigatum, Lapis infernalis mitigatus	Silver nitrate with potassium nitrate should contain no less than 32.3% and no more than 33.5% AgNO_3_ (MW 169.9); the rest is potassium nitrate.	White or greyish-white, odourless, hard sticks with a smooth fracture, very soluble in water.
***Argentum nitricum fusum***	Topiony azotan srebrowy	Argenti nitras fusus	Fused silver nitrate should contain no less than 94.5% and no more than 95.5% AgNO_3_ (MW 169.9); the rest is potassium nitrate.	White or greyish, odorless sticks with a crystalline break. Fused silver nitrate dissolves very easily in water.
***Argentum proteinicum***	Proteinian srebrowy	Argentum proteinatum, Prorgol, Protargol	Silver proteinate should contain no less than 8% and no more than 8.4% Ag (atomic weight 107.91).	Yellow-brown, shiny flakes or yellow-brown, fine powder, almost odorless, with a first bland, then metallic taste. Silver proteinate is easily soluble in water, giving solutions of weakly alkaline reaction.

**Table 5:** Silver preparations described in Therapeutic Guide to the USL (1959), original wording was preserved throughout the table.

The Name of the Preparation by USL	Composition and Counterparts	Action	Application	Usage and Dosage (Doses in Grams)
***Argentum*** ***colloidale subst.***	Contains 70% silver (Argentum) giving colloidal solutions; Colloidal Silver; corgol; collargol	The drug has a bactericidal effect, and mainly stops the development of microorganisms in infectious states, improves the general condition of infections, causes a decrease in temperature.	**Externally**, it is used in inflammation of the mucous membranes (of bacterial origin), the conjunctival sac —in the form of instillations and ointments, and for flushing the urethra and bladder in the form of solutions. It is rarely used **internally**; in general infection and in infectious endocarditis, in typhoid fever.	It is used **externally** for instillation into the conjunctival sac in the form of 0.5–1% solutions, and in ointments 1–15%. For flushing the urethra and bladder, a 0.2–1% solution is used. **Externally** mainly in The form of rubs (*Ung. Credé*), intravenous injections and solutions.
***Argentum*** ***nitricum subst.***	Lapis infernalis, Argenti nitras, silver nitrate, hell stone	Internally, it has an astringent and antispasmodic effect. Externally: strong bactericide, astringent and caustic.	**Externally**, it is used to cauterize the diseased tissue. The drug is also used in inflammation of the mucous membranes (conjunctival sac, stomach, bladder). **Internally**, it is used in chronic diarrhea, peptic ulcer disease, atherosclerosis, in progressive paralysis (rare).	For instillation into the conjunctival sac, 0.25–0.5% solutions are used—after instillation, rinse with saline (0.9% Natrium chloratum aqueous solution). For flushing the bladder, solutions of 1:1000–1:3000 are used—after rinsing, rinse with saline. Internally: 0.01–0.1% solution 3 times a day a tablespoon or in pills (0.01–0.1) per day.
***Argentum*** ***proteinicum subst.***	Contains 8% organically bound silver (Argentum); Silver proteinate; prorgol; protargol	It has a bactericidal effect and anti-inflammatory, in inflammation of the mucous membranes of infectious origin.	It is used in inflammation of the mucous membranes, conjunctival sac—in the form of instillations and ointments, and for flushing the urethra and bladder in the form of solutions. Too much in ulcerations, in infectious inflammations of the nasal mucosa. **Internally** very rarely used in enteritis in children.	It is used **externally** for instillation into the conjunctival sac in the form of 0.5–1% solutions and in ointments 1–15%. For flushing the urethra and bladder—0.2–1%–2% solutions are used. **Internally** 0.1–0.3 per day.

**Table 6:** Doses (concentrations) of preparations according to FP XII.

Name According to the Monograph FP XII	Polish Name	Names in Other Languages (According to Ph.Eur.)	Route of Administration	Doses, Concentrations Typically Used
***Argenti nitras***	Azotan srebra	Silver nitrate; Argent (nitrate d’)	externally	For the skin 1.0–2.0% For flushing 0.05–0.1% Into the conjunctival sac 0.1–0.5% Newborns 1.0% 1 drop into the conjunctival sac For brushing the oral cavity 1.0–2.0% In dentistry 10.0–20.0%
***Argentum colloidale*** ***ad usum externum***	Srebro koloidalne do użytku zewnętrznego	Silver, colloidal, for external use; Argent colloïdal pour usage externe	externally	Solution 0.2–1.0% Ointment 1.0–15.0%

## Data Availability

Not applicable.
